# Degradation of Components in Cars Due to Bimetallic Corrosion

**DOI:** 10.3390/ma14123323

**Published:** 2021-06-16

**Authors:** Mária Hagarová, Janette Brezinová, Gabriela Baranová, Ján Viňáš, Pavlo Maruschak

**Affiliations:** 1 Institute of Materials and Quality Engineering, Faculty of Materials, Metallurgy and Recycling, Technical University of Košice, Letná 9, 042 00 Košice, Slovakia; Gabriela.Baranova@tuke.sk; 2 Department of Technologies, Materials and Computer Aided Production, Faculty of Mechanical Engineering, Technical University of Košice, Mäsiarska 74, 040 01 Košice, Slovakia; Janette.Brezinova@tuke.sk (J.B.); jan.vinas@tuke.sk (J.V.); 3 Department of Industrial Automation, Ternopil Ivan Puluj National Technical University, 46001 Ternopil, Ukraine; maruschak.tu.edu@gmail.com

**Keywords:** bimetallic corrosion, degradation, automotive, surface, corrosion potential

## Abstract

This paper deals with the determination of the basic corrosion characteristics of metallic materials used as components in car construction to achieve a lighter vehicle with higher rigidity, a more complex “hybrid” of diverse materials is needed for the car body structure. Due to the different types of material used in the manufacture of components and their interactions, the issue of assessing the impact of bimetallic corrosion is currently relevant. Based on the potential difference at the end of the corrosion test, it was possible to determine the “anode index”, which determines the risk of degradation of materials due to bimetallic corrosion. In our case, a hot-galvanized steel sheet/Al alloy EN AW-6060 couple in deicing salt and hot-galvanized steel sheet/steel S355J0 couple in simulated acid rain solution (SARS) has proven to be “safest” and usable even for more aggressive environments. Hot-galvanized steel sheet/Al alloy EN AW-6060 in SARS solution is suitable for slightly aggressive environments. Stainless steel AISI 304/silumin A356 in deicing salt, stainless steel AISI 304/Al alloy EN AW-6060 in deicing salt, and stainless steel AISI 306/Al alloy EN AW-6060 in simulated exhaust gas environment (SEG solution) are not suitable for non-aggressive environments.

## 1. Introduction

The automotive industry is a fast-changing, dynamic and innovative industry. For this reason, automotive development requires high-quality, cost-effective and reproducible solutions. Several recent market withdrawals involving Mazda, Toyota, Kia and Mitsubishi were due to the corrosion problems of suspension parts [1]. Water, along with dirt, can enter the ball joint fittings and initiate corrosion, which can cause the front lower control arm to separate from the vehicle. This would make it more difficult to drive, thus increasing the risk of an accident [2]. Problems can occur when joining different materials. When welding Al and steel to reduce the weight of a vehicle, at the point of direct contact of these two different metals in the atmosphere conditions for bimetallic and crevice corrosion are created [3,4]. At present, vehicles are made of diverse metallic materials: aluminum engine blocks and suspension parts, steel car bodies, aluminum dashboard poles. During the final vehicle assembly, these components are attached to a predominantly steel body (steel BIW). To achieve an even lighter vehicle with higher rigidity, a more complex “hybrid” of diverse materials is needed for the car body structure. Bimetallic corrosion in automobiles can occur when the metal comes into contact with a non-metallic conductor, such as carbon in mounting sleeves, or when the metal gets in contact with a different metal, or two different metals come into electrical contact. One metal may corrode preferably to the other. An example is the corrosion of nuts and bolts used to interconnect components [5,6,7]. There are still some concerns with current joining techniques. For example, the joining bit (AISI 4140) form a galvanic corrosion couple in contact with the DP steel surface, as well as in contact with the Al alloy. As study [8] suggests, for automotive applications the joint strength in a corrosive environment should be critically assessed before introducing any combination of different metals, since aluminum alloys generally have a higher electrode potential than steels, and thus aluminum alloys degrade faster than steel. In electric vehicles, bimetallic corrosion between the piston and the cylinder wall can occur due to water absorption in the brake fluid, which causes pitting on the cylinder walls, similarly development of leaks and corrosion formation on the cylinder walls [8,9]. The corrosion problem also occurs in cars in hybrid and fuel-cell versions, where aluminum batteries degrade rapidly due to the formation of aluminum hydroxide [10,11].

There is a wide variety of factors that affect the greatness (intensity) of bimetallic corrosion, such as the area ratio of conductively joined metals, temperature, flow rate, electrolyte composition, and the like. The current between two contacting metals shifts the anode potential to more electropositive value and the cathode potential to more electronegative value. These changes are called polarization. The polarization degree depends on the metal and environment. The extent of polarization determines how effective the specific metal can be as a cathode. The consequence of a change in polarity can be serious. In the zinc-steel and aluminum-steel couples, zinc and aluminum serve as sacrificed anodes to protect the steel. The change in polarity results in the steel cathodic protection loss, it causes galvanic corrosion of the steel and shortens the life of steel structures [12]. Three mechanisms were investigated in the paper [13]: 1. Depletion of dissolved oxygen in the process of oxygen reduction at the stainless-steel cathode and suppression of further aluminum oxidation. 2. Increased Cl^−^ concentration in the electrolyte changed the oxide from stainless steel and impaired its ability to catalyze the oxygen reduction, and thus suppressed the aluminum oxidation. 3. Chemical reactions involving hydrated metal cations that formed surface deposits at active corrosion sites and disrupted further aluminum oxidation. Stainless steels are prone to this type of corrosion, especially if they are in contact with aluminum or low carbon steel.

The contact metals area ratio is very important in terms of the probability of the bimetallic corrosion occurrence. If the galvanic cell is under cathodic control, changes in the anode have little effect on the overall corrosion rate, but changes in the cathode region have a significant effect. The opposite is true if the system is under anodic control. Galvanic currents are in many situations proportional to the surface cathode area; the galvanic zinc corrosion increases with the increasing steel cathode area. On the other hand, the galvanic zinc corrosion changes only very slightly with the increasing zinc anode area. These results suggest that the galvanic zinc corrosion in the system is mainly controlled by the cathode [12,14].

The polarization measurement methods based on electrochemical concepts enable us to determine the instantaneous corrosion rate at the electrode/solution interface by determining the exchange current density in the electrode equilibrium state and the corrosion current density in the corrosion system [15,16].

The risk of formation and extent of bimetallic joint of different metals can be assumed based on the “anode index” by using potentiostatic measurements in different environments. The corrosion potentials of the individual metals for determining the anode index are determined by measuring the steady state open corrosion potential (OCP) vs. the SCE electrode. According to [17], the galvanic compatibility of bimetallic cell components according to the “anode index” is determined depending on the applied environment from the value of the electrode potential difference of the anode and cathode as follows:

- for an aggressive environment, the difference of <150 mV is allowed;
- for a slightly aggressive environment, the difference of <250 mV is allowed;
- for a non-aggressive environment, the difference of <500 mV is allowed.

The risk of a bimetallic joint can also be determined by measuring the polarization diagrams of selected couples of test metallic materials according to Evans. Both metals corrode according to the shape of the polarization curves under non-contact conditions, each at a different rate, and their corrosion potentials E_corr_ (Me) and E_corr_ (Mt) correspond to the equality of the anode and cathode currents. In the case of mutual contact of these metals, the corrosion process will be determined mainly by the cathodic reaction of the more noble of the metal couple, Me, and by the anodic reaction of the less noble metal, Mt. The result will be an increase in current density, and thus corrosion on less noble metal. The main purpose of this study was to determine the bimetallic corrosion risk of several bimetallic couples in three different model environments. The risk of corrosion of a bimetallic couple was determined by measuring the corrosion potential. After long-term exposure in a corrosive environment, the corrosion rate of bimetallic couples was determined by using the Evans and Tafel methods. Depending on the ratio of the anode and cathode area in the bimetallic corrosion cell, the change in the corrosion rate of the two joined metals with different electrochemical zero values was predicted.

## 2. Materials and Methods

### 2.1. Materials and Test Environments

The following materials were used for the experiment: hot-dip galvanized steel (99.3 wt % Zn in the coating), aluminum alloy EN AW-6060, silumin (AlSi_7_Mg_0.3_) A356, stainless steel AISI 304, and steel S355J0. The chemical composition of the tested materials is given in Table 1. The actual chemical composition was determined by atomic emission spectroscopy (AES), infrared absorption, energy-dispersive X-ray spectroscopy (EDX) analysis using an EDX analyzer on a scanning electron microscope JEOL JSM 7000F (Tokyo, Japan).

The thickness of zinc coating (hot-dip galvanized steel samples) was determined by X-ray fluorescence spectrometry (XRF), the thickness was approx. 100 g m^−2^.

Four couples of materials were used for exposure in three different environments, as shown in Table 2.

Model environments were used for the exposure of the tested materials, as shown in the Table 3. The selection was based on a study of the literature, which describes the possibilities of the formation of a bimetallic corrosion cell in a real construction of a car [14,17,18,19,20,21,22,23].

The hot-dip galvanized steel samples were cut from a steel sheet.

The samples were dimensioned 45 × 20 × 2 mm^3^. The samples were cleaned in methanol before exposure. The edges of the samples were insulated with wax.

Table 4 shows combinations of tested bimetallic couples and test environments.

### 2.2. Measurement of Basic Corrosion Characteristics

The following laboratory tests have been carried out to determine the corrosion characteristics of the samples in electrochemical corrosion conditions:

- measurement of the open corrosion potential E_SCE_ samples during their exposure in the environment;
- determination of the kinetics of corrosion of the exposed samples according to Tafel and Stern;
- determination of corrosion potential and current density using Evans polarization diagrams.

#### 2.2.1. Measurement of the Open Corrosion Potential E_SCE_

The open corrosive potential OCP of the test samples was measured against the saturated calomel electrode (SCE) on the Agilent 34405 A 5 1/2 Digit Multimeter (Santa Clara, CA, USA). Corrosion potential expresses the resistance of the surface of the material to its degradation in an aggressive environment. Changes in potential towards more positive values indicate the emergence of a protective layer of corrosion products that protects the surface from further corrosion attack (i.e., a passive layer has formed) [16].

The individual couples of materials were connected to the measuring cells and placed in the environments, Table 1. The corrosion potential was measured for 119 days, while on the first day the measurements were performed at time intervals: 0, 2, 4 and 6 h.

#### 2.2.2. Potentiodynamic Measurements

**Figure 1:**
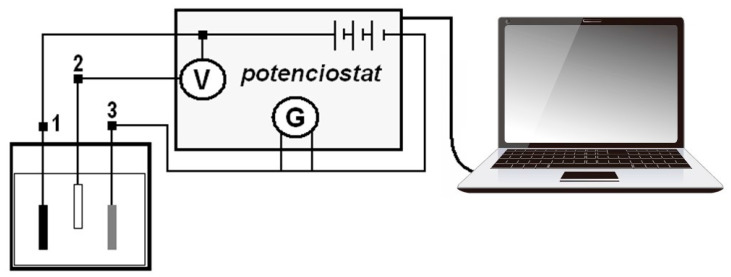
Wiring scheme of a device for measuring linear polarization (1—sample; 2—reference electrode; 3—auxiliary electrode).

The measurement of polarization potentiodynamic curves was performed on a Voltalab PGP 201 instrument (Loveland, CO, USA) with Voltamaster 4 software, Figure 1. The sample was polarized from an external source with direct current in a selected range of potentials, and the current density required to reach these potentials was determined. The corrosion rate depends on the kinetics of anode (oxidation) and cathode (reduction) reactions, while the calculation of the rate of corrosion attack is based on the determination of corrosion currents, determined by measuring [15,16]. The polarization resistance Rp was determined from the Stern polarization curve. Measurements according to Tafel and Stern were performed in the 1st and last 18th week of sample exposure.

To determine the Evans polarization diagrams, measurements were performed on a Voltalab PGP 201 instrument with Voltamaster4 software. The measurement of Evans polarization diagrams uses a three-electrode connection: working electrode (sample from the tested couple with a more negative free potential), auxiliary electrode (sample with a more positive free potential), reference electrode (saturated calomel electrode). Measurements of Evans polarization diagrams were performed at weeks 4, 9, 14, and 18 of sample exposure.

## 3. Results and Discussion

### 3.1. Measurement of the Open Corrosion Potential of E_SCE_ Samples during Their Exposure in the Environment

**Figure 2:**
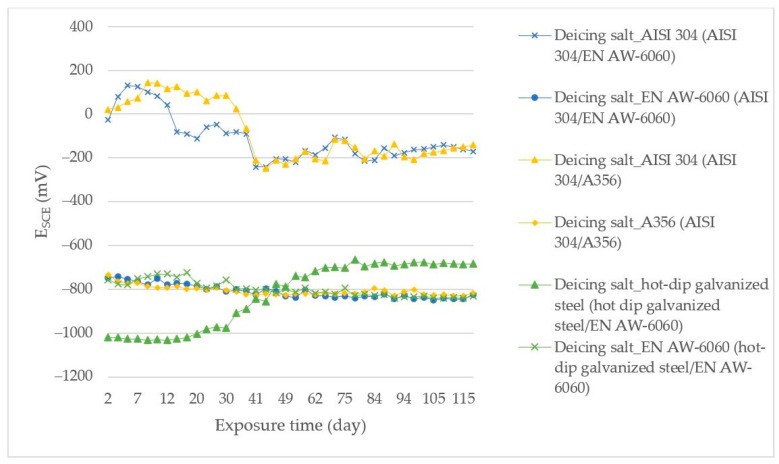
E_SCE_ measurement results for bimetallic couples exposed in deicing salt.

Figure 2 shows the corrosion potential measured time courses of the couples exposed in deicing salt (3% NaCl).

Based on the measured time courses of the corrosion potentials, which were graphically processed in Figure 2, the E_SCE_ potential of AISI 304 (in bimetallic joint with EN AW-6060) exposed in deicing salt (3% NaCl) increased towards more positive values during the first 11 days of the test, to max E_SCE_ = +130 mV. During that period, a passive layer formed on the steel surface, which dissolved in deicing salt during the next exposure of the steel. This change was accompanied by a decrease in the corrosion potential to more negative values; up to the final value −170 mV. The whole course of AISI 304 corrosion degradation resulted in a relatively large change in the corrosion potential, namely 300 mV during the whole measurement period. EN AW-6060 (in a bimetallic joint with AISI 304) exposed in deicing salt had a steady E_SCE_ potential course, which after 28 days of testing reached more negative values than −800 mV, with a final E_SCE_ = −824 mV. This fact indicates that no passive layer was formed in the chloride environment.

The AISI 304 potential (in a joint with EN AW-6060) in deicing salt rose to more positive values up to the 34th day, when it dropped to negative values below −150 mV, followed by the passive layer dissolution that had formed on the sample surface during the first third of the test. This drop occurred 20 days later than in the case of EN AW-6060 and AISI 304 combination. The potential difference ΔE during the measurement was max 393 mV. The A356 (in the bimetallic joint with AISI 304) in deicing salt had a steady course of the E_SCE_ potential, which fluctuated continuously towards more negative values, from −745 mV at the beginning of measurement to −819 mV at the end of measurement.

Hot-dip galvanized steel (in combination with EN AW-6060) in deicing salt had at the beginning of the corrosion test potential higher than −1000 mV, specifically −1035 mV. After the 21st day of exposure, it changed to less negative values. At the end of the test, the E_SCE_ was about −680 mV. The EN AW-6060 corrosion potential (in the bimetallic joint with A356) in deicing salt had a similar course during the test as the EN AW-6060 potential in a bimetallic joint with AISI 304 (small deviations represented a potential difference of max 60 mV, this difference changing in a short time). At the end of the exposure, E_SCE_ = −831 mV was measured.

**Figure 3:**
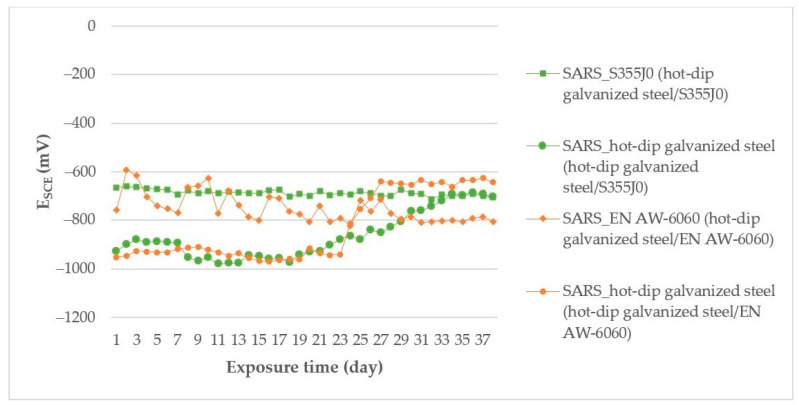
E_SCE_ measurement results for bimetallic couples exposed in SARS.

Figure 3 shows the corrosion potential measured time courses of the couples exposed in SARS.

Based on the measured time courses of the corrosion potentials (Figure 3), the hot-dip galvanized steel (in the bimetallic couple with S355J0) in SARS had a stabilized potential course below −950 mV on the first day of the test, which after the second day began to rise to more positive values above −890 mV, up to the 12th day of the test. From here, until the 30th day of the test, the hot-dip galvanized steel sample potential decreased to values below −950 mV, from where it started to rise again to more positive values until it reached the final value of −704 mV. The S355J0 corrosion potential (in connection with hot-dip galvanized steel) in the SARS environment on the first day of measurement decreased from the value E_SCE_ = −382 mV to E_SCE_ = −615 mV. This decrease in potential was recorded until the eighth day of measurement when it stabilized below −670 mV and up to the final value of −704 mV. The hot-dip galvanized steel sample (in the bimetallic joint with EN AW-6060) in SARS solution showed a similar potential profile as the hot-dip galvanized steel in the couple with steel S355J0; thus, on the first day of the test, the potential course was stabilized below −950 mV. However, from the second day of the test, the corrosion potential began to rise towards more positive values until the 16th day, dropping to a value of −969 mV on the 41st day of the test. From that day on, there was an increase in the potential, which stabilized at a value below −640 mV. EN AW-6060 (in the bimetallic joint with hot-dip galvanized steel) in SARS environment showed on the first day of measurement a potential drop from the value −568 mV to the value −890 mV, which subsequently rose again to value −579 mV. This alternation of decreases and increases of E_SCE_ was recorded until the 92nd day of measurement, when the potential values stabilized below −800 mV. Such alternation of potentials indicates passive layer formation and subsequent dissolution on the EN AW-6060 surface in joint with hot-dip galvanized steel.

**Figure 4:**
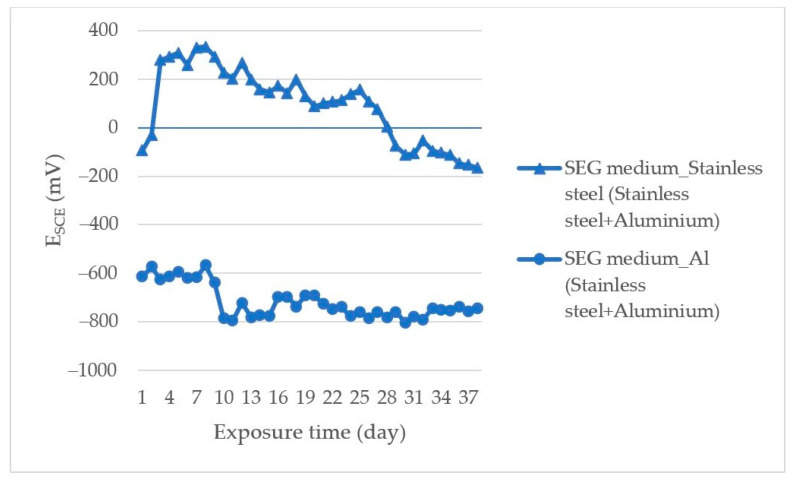
E_SCE_ measurement results for stainless steel/Al couple exposed in SEG.

Figure 4 shows the corrosion potential measured time courses of the AISI 304/EN AW-6060 couple exposed in SEG.

Based on the measured time courses of the corrosion potentials (Figure 4), AISI 304 (in a bimetallic joint with EN AW-6060) exposed in SEG solution had potential in positive values; max E_SCE_ = +334 mV until the 84th day of the test when it dropped to the final value −164 mV. This decrease indicated dissolution of the corrosion products layer formed on the steel surface. The difference in corrosion potential ΔE caused by the action of the corrosive environment on the steel surface was 498 mV at the end of measurement. The EN AW-6060 corrosion potential (in the couple with AISI 304) in SEG solution on the first day of measurement after 6 h slightly increased to the value −567 mV and in this potential range it stayed up to the 16th day of measurement, while from the 16th day of the test the potential decreased to the min value E_SCE_ = −805 mV.

The risk of formation and the extent of bimetallic corrosion of different metals can be assumed based on the “anode index” [15,16]. The open corrosion potential (OCP) measurement results are summarized in Table 5.

The hot-dip galvanized steel/EN AW-6060 couple in SARS solution, the AISI 304/EN AW-6060 couple in SEG solution and, the AISI 304/A356 in deicing salt created secure joints for the given environment.

### 3.2. Determination of the Exposed Samples Corrosion Kinetics according to Tafel and Stern

**Figure 5:**
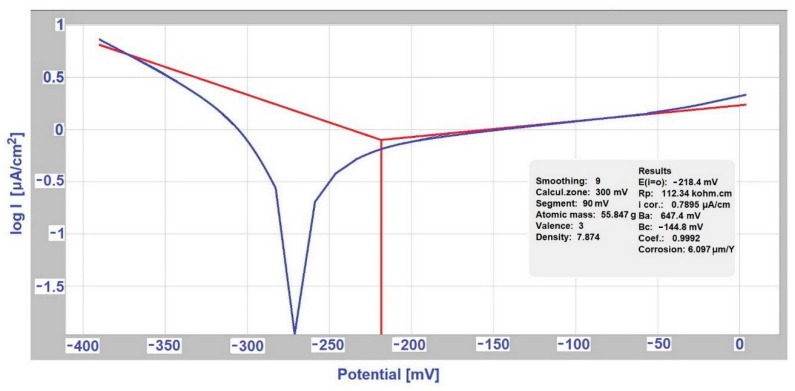
Measurement according to Tafel for stainless steel (hot-dip galvanized steel/EN AW-6060 couple) in the first week (deicing salt).

**Figure 6:**
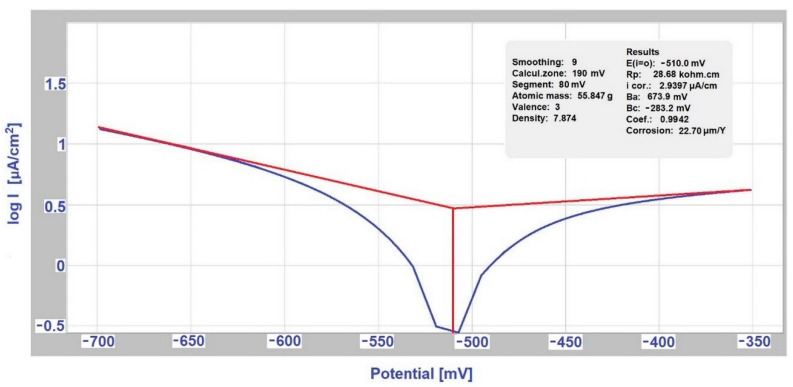
Measurement according to Tafel for stainless steel (hot-dip galvanized steel/EN AW-6060 couple) in the 18th week (deicing salt).

As for other bimetallic couples, a several times higher rate of metal dissolution was observed in the case of a conductive connection with another metal in a given environment compared to the value of dissolution of the metal itself. The corrosion characteristics of individual samples were measured by the linear polarization method, namely corrosion current J_corr_ (μA·cm^−2^), polarization resistance R_p_ (kΩ·cm^2^). Their values are given in Table 3. Graphic records from the Voltamaster4 software in the first week of selected measurement are shown in Figure 5 and the measurement records from the 18th week are shown in Figure 6.

These measurements at the beginning and end of the corrosion test were used to determine the rate and nature of changes that occurred on the exposed samples surface. The corrosion current per unit area represents the corrosion rate, i.e., metal dissolution rate. The formation of corrosive products can create a protective barrier, which can slow down, stops the dissolution of the metal. The barrier effectiveness is reflected in the increase of the polarization resistance R_p_. The measured values are shown in Table 6.

For AISI 304/EN AW-6060 bimetallic couple, and respectively AISI 304/A356 (in deicing salt), the polarization resistance R_p_ of the steel decreased and the corrosion rate increased. R_p_ drop indicated that no passive layer was formed on the steel surface.

For hot-dip galvanized steel/EN AW-6060 couple (in the deicing salt environment), hot-dip galvanized steel/EN AW-6060 joint (in SARS environment) and Zn hot-dip galvanized steel/S355J0 joint (in SARS environment), during exposure on the surface a layer of corrosion products was formed, which led to a significant increase in R_p_ after the 18th week of the test, namely: from 0.04032 kΩ·cm^2^ to 8.85 kΩ·cm^2^ for hot-dip galvanized steel/EN AW-6060 couple in deicing salt; from 3.48 kΩ·cm^2^ to 7.2 kΩ·cm^2^ for hot-dip galvanized steel/S355J0 couple in SARS and from 3.48 kΩ·cm^2^ to 8.53 kΩ·cm^2^ for hot-dip galvanized steel/EN AW-6060 joint in SARS.

For AISI 304/EN AW-6060 couple in SEG solution, there was a decrease in the corrosion current on stainless steel, which shows the opposite effect as shown by stainless steel in combination with Al alloy in deicing salt.

### 3.3. Determination of Corrosion Potential and Current Density Using Evans Polarization Diagrams

**Figure 7:**
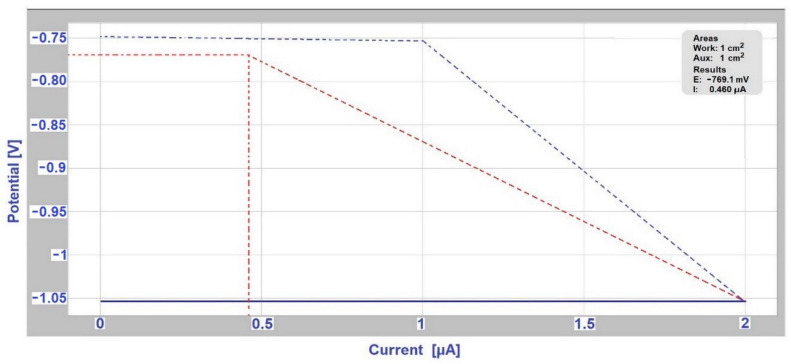
Evans diagram for hot-dip galvanized steel/EN AW-6060 couple (ratio of anode A and cathode A:C is 1:1) in deicing salt in the fourth week of measurement.

**Figure 8:**
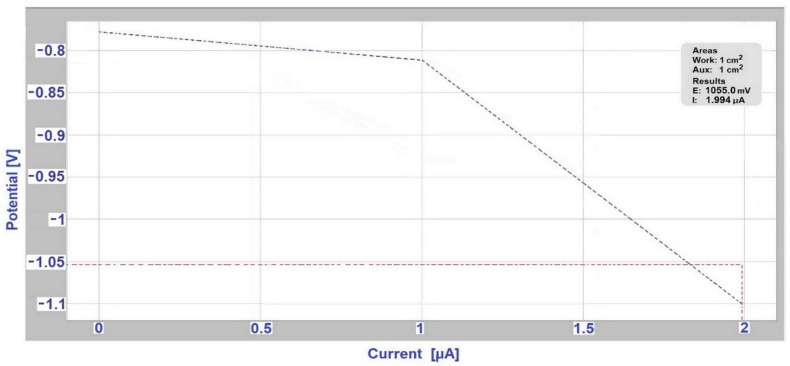
Evans diagram for hot-dip galvanized steel/EN AW-6060 couple (ratio of anode A and cathode A:C is 1:1) in deicing salt in the ninth week of measurement.

The results from the measurement of Evans polarization diagrams are shown in Table 6, which also predicts the increase in current for different ratios of anode areas to the cathode area. From these results it is obvious that a connection was formed between the individual couples of metals, which was caused by the conductive contact of individual metals using the electrolyte. For the ratio of anode area and cathode area 1:1, the graphical measurement outputs from the Voltamaster4 software for the selected hot-dip galvanized steel/EN AW-6060 couple in deicing salt in the fourth week of the test are shown in Figure 7 and in the ninth week of the test in Figure 8. The ratio of anode (A) area and cathode (C) area A:C affects the magnitude of the current density in the bimetallic cell. The corrosive current flows from the anode, and if the anode A is several times smaller than the cathode area C, the current is concentrated on a relatively small area, and the current density increases significantly. For AISI 304/EN AW-6060 couple (in deicing salt), the maximum corrosion current value J_corr_ increased from 27.797 μA to 35.982 μA, and then the corrosion current decreased to a final value 20.151 μA for the area A:C = 1:1. For stainless steel/Al alloy (in the SEG solution) couple and hot-dip galvanized steel/S355J0 (in SARS solution) couple, there was a decrease in corrosion current for the area A:C = 1:1 in the ninth week of measurement (compared to the fourth week of the test); in the 14th week an increase was observed and in the 18th week of measurement the value dropped to the corrosion current final value 4.845 μA for AISI 304/EN AW-6060, and respectively 59.570 μA for hot-dip galvanized steel/S355J0 couple. For AISI 304/A356 couple (in deicing salt) the maximum corrosion current value J_max_ increased from 41.700 μA to 44.207 μA, followed by a drop and finally again the corrosion current increased for the 1:1 area to 40.349 μA.

The hot-dip galvanized steel/EN AW-6060 couple (in deicing salt) showed an increase in corrosion current during the whole measurement period, from the value J_max_ = 0.460 μA in the fourth week to 24.321 μA in the 18th week.

Table 7 shows a comparison of current density values that define the anode dissolution in a bimetallic joint, which were obtained from Evans polarization diagrams for surfaces A:C = 1:1. J_corr_ values were obtained by the Tafel method after 18 weeks of exposure in deicing salt.

The hot-dip galvanized steel/EN AW-6060 couple (in SARS solution), the AISI 304/EN AW-6060 couple (in SEG solution) and the AISI 304/A356 (in deicing salt) formed secure joints for the given environment. For other bimetallic couples, a several times higher metal dissolution rate was measured for conductive connection with another metal in a given environment compared to the dissolution value of the metal itself.

## 4. Conclusions

The tests performed enabled us to determine the bimetallic corrosion risk of diverse metals forming bimetallic couples, in SARS, deicing salt, and SEG test environments.

The surface passivation of aluminum-based alloys led to a stable course of E_SCE_ in deicing salt. For EN AW-6060 in AISI 304/EN AW-6060 couple, A356 in AISI 304/A356 couple, and EN AW-6060 in hot-dip galvanized steel/EN AW-6060 couple, the E_SCE_ corrosion potential had a slight course throughout the exposure time which corresponded with ΔE in the interval 75–85 mV.

For AISI 304 in AISI 304/EN AW-6060 couple and AISI 304/A356 couple, the onset of steady-state was noticed after 40 days of exposure, when the E_SCE_ reached −242 mV, respectively −211 mV. By the end of the exposure time, the course of E_SCE_ was already slight (stable), with the difference ΔE = 72 mV for AISI 304 in AISI 304/EN AW-6060, resp. ΔE = 70 mV.

For hot-dip galvanized steel in hot-dip galvanized steel/EN AW-6060, the zinc coating dissolved, while the E_SCE_ value in the second half time of the test gradually approached the near E_SCE_ values of the steel, but remained stabilized, specifically reaching the E_SCE_ = −684 mV at the end of the test.

The previous statement was also confirmed for the hot-dip galvanized steel/S355J0 408 couple in SARS. The potential of E_SCE_ hot-dip galvanized steel increased due to the zinc coating dissolution. The corrosion potential course of the measured couples was relatively moderate in SARS, with the smallest difference ΔE = 41 mV for S355J0 in the couple with hot-dip galvanized steel measured in the E_SCE_ interval in negative values. The most negative initial potential values were determined on hot-dip galvanized sheets in the couple with EN AW-6060, namely −951 mV, which during the test were shifting towards less negative values to reach the final value E_SCE_ = −643 mV.

In SEG, the corrosion potential of AISI 304 in contact with EN AW-6060 showed positive values for 2/3 of the test with max E_SCE_ = 334 mV. After 85 days of testing (out of a total of 119 days), it reached a negative value with a drop to the final E_SCE_ = −164 mV. The corrosion potential of EN AW-6060 in the couple with AISI 304 showed an initial value of −615 mV, and ΔE = 129 mV was observed for the final value of −744 mV during the test.

Based on the ΔE potential differences, the risk of bimetallic corrosion was determined:

- bimetallic couples of hot-dip galvanized steel/EN AW-6060 in deicing salt and hot-dip galvanized steel/S355J0 in SARS proved to be the safest and most usable even for more aggressive environments;
- bimetallic couple of hot-dip galvanized steel/EN AW-6060 in SARS solution is suitable for slightly aggressive environments;
- bimetallic couple of AISI 304/EN AW-6060 in deicing salt and SEG, and AISI 304/A356 couples in deicing salt represent the risk of bimetallic corrosion even in a non-aggressive environment.

For bimetallic couples, a several times higher rate of metal dissolution was measured for conductive connection with another metal in a corrosive environment compared to the dissolution value of the metal itself. This was also confirmed based on measured values of current densities in all cases, except:

- hot-dip galvanized steel/EN AW-6060 in SARS solution;
- AISI 304/EN AW-6060 in SEG;
- A356/AISI 304 in deicing salt.

## Figures and Tables

**Table 1:** Chemical composition of used materials (in wt %).

Element	Hot-Dip Galvanized Steel—Substrate	S255J0	EN AW-6060	AISI 304	AlSiMg0.3
C	0.0024	0.024	-	0.05	-
Mn	0.140	0.470	0.10	1.65	0.08
Si	0.007	0.094	0.46	0.84	7.25
Cr	0.009	-	0.05	18.2	-
Ti	0.061	0.025	0.10	-	0.19
S	0.014	0.003	-	0.012	-
P	0.009	0.002	-	0.034	-
N	0.002	0.002	-	-	-
Nb	<0.002	0.0072	-	-	-
Cu	0.028	-	0.11	-	0.01
Mg	-	-	0.48	-	0.42
Sr	-	-	-	-	0.012
Ni	-	-	-	8.61	-
Zn	-	-	0.15	-	0.01
Al	-	0.042	bal.	-	bal.
Fe	-	bal.	0.37	bal.	0.09

**Table 2:** Model environment for testing material couples.

Environment	Chemical Composition in MmoL L^−1^	Model Environment for:
SARS (simulated acid rain solution)	0.01 mmoL L^−1^ HNO_3_; 1.0 mmoL L^−1^ NaCl; 1.0 mmoL L^−1^ (NH_4_)_2_SO_4_	industrial environment
deicing salt	3 % NaCl	deicing salt
SEG (simulated exhaust gas environment)	52.1 mmoL L^−1^ (NH_4_)_2_SO_4_; 2.8 mmoL L^−1^ NH_4_Cl; 1.6 mmoL L^−1^ NH_4_NO_3_; 2.2 mmoL L^−1^ HCOOH	condensed exhaust gas

**Table 3:** Couples of material samples assembled based on real joints in car construction.

Bimetallic Couple	Part of the Car Construction	Resource
hot-dip galvanized steel/EN AW-6060	roof, hood, or doors	Meschut G.; Janzen, V.; Olfermann, T., 2014
AISI 304/EN AW-6060	EASW (Element Arc Spot Welding) mechanism rivers, screws/Al sheet layers of steel with aluminum bars in the rotor	Naito, J.; Suzuki, R. 2020 Mori, K., Abe, Y., 2018
AISI 304/A356	exhaust systems (catalytic converter „cat „) oil pan, valve cover, hera gasket/cylinder head	Leitman, S., Brant, 2009
hot-dip galvanized steel/S355J0	body plates/roof suspension system components/shock absorbers	Shibli, S. M. A.; Meena, B. N.; Remya, R., 2015 Li, G., Long, X., 2020

**Table 4:** Couples of materials in model environments (x—tested).

Sample Couples	Environment		
	SARS	Deicing Salt	SEG Solution
hot-dip galvanized steel/EN AW-6060	X	X	
AISI 304/EN AW-6060		X	X
AISI 304/A356		X	
hot-dip galvanized steel/S355J0	X		

**Table 5:** Usability of bimetallic corrosion of different metals in the environment.

Anode Index According to [15,16]	Environment	Usability of Couple of Metals in Environment	Anode Index According to the Measurement E_SCE_	Tested Couple
ΔE < 500 mV	Non-aggressive environment	not	ΔE = 678 mV	AISI 304/A356 in deicing salt
		not	ΔE = 654 mV	AISI 304/EN AW-6060 in deicing salt
		not	ΔE = 580 mV	AISI 304/EN AW-6060 in SEG
ΔE < 250 mV	slightly environment	yes	ΔE = 161 mV	hot-dip galvanized steel/EN AW-6060 in SARS
ΔE < 150 mV	aggressive environment	yes	ΔE = 147 mV	hot-dip galvanized steel/EN AW-6060 in deicing salt
		yes	ΔE = 2 mV	hot-dip galvanized steel /S355J0 in SARS

**Table 6:** Measurement according to Tafel and Stern.

Environment	Sample	Measurement (1st Week)		Measurement (18th Week)	
		J_corr_ (μA·cm^−2^)	Rp (kΩ·cm^2^)	J_corr_ (μA·cm^−2^)	Rp (kΩ·cm^2^)
deicing salt	AISI 304	0.79	60.85	2.9397	21.2
	EN AW-6060	0.0636	1380	0.6896	61.58
SEG solution	AISI 304	22.7	4.02	10.9742	3.99
	EN AW-6060	0.1426	396.6	4.6835	8.34
deicing salt	AISI 304	0.79	60.85	3.4158	15.7
	A356	38.37	1.91	76.5935	0.50216
SARS	hot-dip galvanized steel	23.378	3.48	6.1731	7.2
	S355J0	0.4489	50.08	45.7841	1.12
SARS	hot-dip galvanized steel	23.378	3.48	5.8018	8.53
	EN AW-6060	0.146	435.58	0.144	434.93
deicing salt	hot-dip galvanized steel	2268.3	0.04032	2.1851	8.85
	EN AW-6060	0.0636	1380	0.3173	14.8

**Table 7:** Comparison of current density J_max_ measured by Evans and current density J_corr_ measured by Tafel.

Sample	Exhibition	J_max_ (Evans)	J_corr_ (Tafel)
		(μA·cm^−2^)	
hot-dip galvanized steel sheet	separately (exposed with EN AW-6060 sample in deicing)	-	2.1851
	in couple with EN AW-6060 (in deicing salt)	24.321	-
	separately (exposed with S355J0 steel in SARS solution)	-	6.1731
	in couple with S355J0 (in SARS solution)	59.570	-
	separately (exposed to EN AW-6060 sample in SARS solution)	-	5.8018
	in couple with EN AW-6060 (in SARS solution)	0.636	-
Al alloy EN AW-6060	separately (exposed with hot-dip galvanized steel sample in deicing salt)	-	0.3173
	in couple with hot-dip galvanized steel (in deicing salt)	24.321	-
	separately (exposed with AISI 304 sample in deicing salt)	-	0.6896
	in couple with AISI 304 (in deicing salt)	20.151	-
	separately (exposed with AISI 304 in SEG solution)	-	4.6835
	in couple with AISI 304 (in SEG solution)	4.845	-
	separately (exposed with hot-dip galvanized steel in SARS solution)	-	0.144
	in couple with hot-dip galvanized steel (in SARS solution)	0.636	-
stainless steel AISI 304	separately (exposed with EN AW-6060 in deicing salt)	-	2.9397
	in couple with EN AW-6060 (in deicing salt)	20.151	-
	separately (exposed with A356 in deicing salt)	-	3.4158
	in couple with A356 (in deicing salt)	40.349	-
	separately (exposed with EN AW-6060 in SEG solution)	-	10.9742
	in couple with EN AW-6060 (in SEG solution)	4.845	-
silumin A356	separately (exposed with AISI 304 in deicing salt)	-	76.5935
	in couple with AISI 304 (in deicing salt)	40.349	-
steel S355J0	separately (exposed with hot-dip galvanized steel in SARS solution)	-	45.7841
	in couple with hot-dip galvanized steel (in SARS solution)	59.570	-

## Data Availability

The data presented in this study are available upon request from the corresponding author.
